# Group-informed attentive framework for enhanced diabetes mellitus progression prediction

**DOI:** 10.3389/fendo.2024.1388103

**Published:** 2024-06-24

**Authors:** Changting Sheng, Luyao Wang, Caiyi Long, Rensong Yue

**Affiliations:** Hospital of Chengdu University of Traditional Chinese Medicine, Chengdu, Sichuan, China

**Keywords:** diabetes mellitus, cluster, deep attentive transformer, regression, group-level features

## Abstract

The increasing prevalence of Diabetes Mellitus (DM) as a global health concern highlights the paramount importance of accurately predicting its progression. This necessity has propelled the use of deep learning’s advanced analytical and predictive capabilities to the forefront of current research. However, this approach is confronted with significant challenges, notably the prevalence of incomplete data and the need for more robust predictive models. Our research aims to address these critical issues, leveraging deep learning to enhance the precision and reliability of diabetes progression predictions. We address the issue of missing data by first locating individuals with data gaps within specific patient clusters, and then applying targeted imputation strategies for effective data imputation. To enhance the robustness of our model, we implement strategies such as data augmentation and the development of advanced group-level feature analysis. A cornerstone of our approach is the implementation of a deep attentive transformer that is sensitive to group characteristics. This framework excels in processing a wide array of data, including clinical and physical examination information, to accurately predict the progression of DM. Beyond its predictive capabilities, our model is engineered to perform advanced feature selection and reasoning. This is crucial for understanding the impact of both individual and group-level factors on deep models’ predictions, providing invaluable insights into the dynamics of DM progression. Our approach not only marks a significant advancement in the prediction of diabetes progression but also contributes to a deeper understanding of the multifaceted factors influencing this chronic disease, thereby aiding in more effective diabetes management and research.

## 1 Introduction

Diabetes Mellitus (DM) stands as a global health crisis, characterized by its widespread prevalence and significant health hazards Association (1) Alam et al. (2) Tomic et al. (3). The disease’s impact on quality of life and its association with various complications Nathan (4); Cole and Florez (5) underscore the urgency for effective management and intervention strategies. In recent years, the integration of deep learning models in medical diagnostics has shown promising results Litjens et al. (6) Ayon and Islam (7) Liu et al. (8) Pal et al. (9), offering innovative approaches to disease detection and progression prediction. In the realm of diabetes, deep learning techniques have been applied to electronic medical records and hospitalization data for diagnosing and predicting diabetes, prediabetes, and its complications Arcadu et al. (10) Ljubic et al. (11) Refat et al. (12) Gupta et al. (13). Additionally, these methods have seen some success in real-time blood glucose monitoring Zhu et al. (14) Freiburghaus et al. (15, 16).

In this work, we focus on employing deep learning techniques to predict the progression of DM (taking blood glucose concentration as an example) based on common clinical data and physical examination indicators, which can significantly enhance the ability to identify disease risks, thereby enabling early warning for patients. This approach not only aids in timely intervention to reduce the risk of complications but also provides critical information for the formulation of personalized treatment plans, thus improving long-term health outcomes for patients Ljubic et al. (11) Yahyaoui et al. (17). In addition, precise prediction of the trajectory of diabetes allows healthcare providers to allocate resources more effectively and optimize treatment strategies Choi et al. (18) Li et al. (19), ultimately enhancing the quality of life and disease management capabilities for patients.

However, this endeavor faces several critical challenges. First, a key challenge lies in the inevitable occurrence of missing data, as the range of medical tests conducted can vary across different populations. Designing effective methods for data imputation to enhance the model’s ability to cope with data gaps is crucial. Second, the values of individual examination indicators are prone to fluctuation due to changes in diet and lifestyle Pala et al. (20) Du et al. (21). Designing a robust system for DM progression prediction that remains unaffected by minor variations in these indicators is also important. Lastly, utilizing effective and interpretable deep learning models to analyze and explain the impact of various indicators on diabetes progression, as well as the interrelationships among these indicators, is crucial and presents a significant challenge. Such interpretability is essential for providing meaningful guidance to medical and informatics researchers and practitioners. It enables a deeper understanding of the disease mechanisms and supports the development of more targeted and effective diabetes management strategies. Achieving this level of clarity and explanation in model outputs is key to advancing the field and enhancing the practical utility of predictive analytics in healthcare.

In response to the aforementioned challenges, our study makes the following contributions: (*i*) To tackle the problem of missing data, we initially cluster the samples in the dataset based on clinical indicators. Subsequently, we utilize the indicative information from these clusters to enhance the effectiveness of our data imputation algorithm; (*ii*) To improve the stability of the predictive model, we introduce methods of sample augmentation with permutation injection and group-level feature augmentation. These methods aim to minimize the impact of minor fluctuations in indicators on the model and expand the model’s training environment, thereby enhancing its robustness and predictive performance; (*iii*) We leverage a deep attentive transformer, i.e., the TabNet model Arik and Pfister (22), which allows for both static and dynamic analysis of the impact and importance of different indicators on model predictions through feature weights and masks. Additionally, by applying group masking to the inherent grouping of clinical data features, we analyze and interpret the influence of different categories of test indicators on the model. The grouping of clinical data features also indirectly provides the predictive model with information about the interrelationships between features, thereby improving prediction accuracy.

We have opted for blood glucose concentration (BG) as the primary indicator for monitoring the progression of diabetes mellitus (DM), rather than glycated hemoglobin (HbA1c) Sherwani et al. (23). This decision is based on a key consideration: BG provides immediate feedback on glucose levels, which is crucial for acute diabetes management. Our model is designed to capture and respond to rapid changes in glucose levels to prevent and manage acute complications such as hypoglycemia or ketoacidosis. In contrast, HbA1c offers an average blood glucose level over the past two to three months and is better suited for long-term diabetes management and monitoring the risk of chronic complications, rather than for situations requiring immediate decision-making Weykamp (24).

In summary, this work explores methods of DM progression prediction, aiming to reveal the impact of various indicators on the predictive task. It conducts multifaceted analysis and exploration of population and features at the group level. By delving deeper into the interpretation of how different group information influences DM prediction, the study also significantly enhances the model’s predictive capabilities. Detailed individual and group-level analyses are believed to bring new insights to related research. We have named our predictive model the Group-informed Attentive framework for Diabetes Mellitus progression Prediction (GADMP).

## 2 Materials and preliminaries

In this section, we first provide a detailed analysis of the dataset employed in this study, followed by a brief introduction to the deep attentive transformer, which is the primary methodology used^1^.

### 2.1 Dataset

In this study, our dataset is sourced from the public dataset of the 2018 Tianchi Precision Medicine Competition^1^, which is curated to explore the correlation between various common indicators and the progression of diabetes mellitus. Specifically, it facilitates the prediction of the progression of DM in a population (using blood glucose concentration as the key indicator), by analyzing clinical data and physical examination indicators from diabetic patients. Intelligent prediction of blood glucose levels aids in diagnostic support, while an interpretable predictive model, by analyzing the relationships between different indicators and blood glucose levels, can help expand medical and research perspectives. The dataset comprises 6,642 rows and 42 columns, representing 42 attributes for 6,642 patients, including ID, gender, age, date of physical examination, various indicators, and so forth. The last column is filled with blood glucose concentration, which is our target for prediction. BG is measured after an overnight fast and is often used to gauge the effectiveness of diabetes management on a somewhat longer scale.

In **Table 1**, we present a detailed list of the attributes from the dataset used for our prediction task. These attributes are typical of tabular data and can be broadly divided into two categories: categorical features, such as gender, and numerical features, including age and various medical test indicators. We have excluded attributes like user ID and physical examination date from the model building process, as they do not have a direct correlation with health conditions. Apart from basic demographic information like age and gender, the dataset includes 37 medical test indicators. These indicators are further categorized into six groups (*B*-*G*) based on the specific bodily functions they assess, such as liver function tests and lipid profiles. **Table 1** also shows the extent of missing data for each feature. We observe that, besides basic demographic data, all medical indicators have some level of missing data, with the number of patients missing these data ranging from 21 (e.g., WBC) to 5110 (e.g., HBsAg). The presence of missing data in this dataset highlights a key challenge in our research. That is, how to develop effective algorithms to address widespread data gaps, thereby enabling the creation of a universally applicable diabetes progression prediction model.

**Table 1:** Statistics of clinical data.

Group (Group Index)	Attribute Index	Attribute Name	Missing Count
Basic Demographics (*A*)	*A*_1_ *A*_2_	Age Gender	0 0
Liver Function Tests (*B*)	*B*_1_ *B*_2_ *B*_3_ *B*_4_ *B*_5_ *B*_6_ *B*_7_ *B*_8_	Aspartate Aminotransferase (AST) Alanine Aminotransferase (ALT) Alkaline Phosphatase (ALP) Gamma-Glutamyl Transferase (GGT) Total Protein Albumin Globulin Albumin/Globulin Ratio	1406 1406 1406 1406 1406 1406 1406 1406
Kidney Function Tests (*C*)	*C*_1_ *C*_2_ *C*_3_	Urea Creatinine Uric Acid	1572 1572 1572
Lipid Profile (*D*)	*D*_1_ *D*_2_ *D*_3_	Triglycerides (TG) Total Cholesterol (TC) High-Density Lipoprotein Cholesterol (HDL-C)	1395 1395 1395
	*D*_4_	Low-Density Lipoprotein Cholesterol (LDL-C)	1395
Hepatitis B Virus Markers (*E*)	*E*_1_ *E*_2_ *E*_3_	Hepatitis B Surface Antigen (HBsAg) Hepatitis B Surface Antibody (HBsAb) Hepatitis B e Antigen (HBeAg)	5110 5110 5110
	*E*_4_	Hepatitis B e Antibody (HBeAb)	5110
	*E*_5_	Hepatitis B Core Antibody (HBcAb)	5110
Complete Blood Count (*F*)	*F*1 *F*2 *F*3 *F*4 *F*5 *F*6 *F*7	White Blood Cell Count (WBC) Red Blood Cell Count (RBC) Hemoglobin (HGB) Hematocrit (HCT) Mean Corpuscular Volume (MCV) Mean Corpuscular Hemoglobin (MCH) Mean Corpuscular Hemoglobin Concentration (MCHC)	21 21 21 21 21 21 21
	*F*8 *F*9	Red Cell Distribution Width (RDW) Platelet Count	21 21
	*F*10	Mean Platelet Volume (MPV)	29
	*F*11	Platelet Distribution Width (PDW)	29
	*F*12	Plateletcrit (PCT)	29
White Blood Cell Differential Count (*G*)	*G*_1_ * G*_2_ * G*_3_ *G*_4_ *G*_5_	Neutrophils Percentage Lymphocytes Percentage Monocytes Percentage Eosinophils Percentage Basophils Percentage	21 21 21 21 21

### 2.2 Modeling tabular data

In the realm of tabular data processing for predictive tasks (regression or classification), traditional approaches have predominantly relied on tree-based ensemble learning algorithms, such as Random Forest Svetnik et al. (25), Gradient Boosting Machines (GBM) Friedman (26), XGBoost Chen and Guestrin (27), and LightGBM Ke et al. (28). These algorithms excel with structured data due to their effective handling of non-linear relationships between features Nguyen and Byeon (29). Additionally, tree-based models are generally more interpretable, a quality of significant importance in various business and decision-making contexts. In contrast, deep learning algorithms are more adept at managing large-scale unstructured data Shwartz-Ziv and Armon (30), such as images Shen et al. (31), text Chatterjee et al. (32), and audio Purwins et al. (33). Deep learning models are capable of autonomously extracting complex, hierarchical features, which is particularly crucial when dealing with intricate data types like pixel data or natural language.

In recent years, with the advent of the TabNet model Arik and Pfister (22), the application of deep learning in processing tabular data has seen a notable increase Li et al. (19) Yan et al. (34) Chen et al. (35). TabNet, a deep learning model specifically designed for tabular data, stands out for its integration of the Attentive Transformer and significant interpretability. The Attentive Transformer enables TabNet to dynamically select and focus on the most crucial input features, thereby enhancing predictive performance and the ability to handle complex datasets. Furthermore, TabNet’s design emphasizes model interpretability, primarily due to its dynamic feature selection and attention mask mechanism. This allows for effective identification and utilization of the most predictive features, improving both the model’s performance and interpretability. Such capabilities are particularly vital for applications requiring high levels of transparency and interpretability.

However, the direct application of the existing standard TabNet model to predict blood glucose concentration encounters several practical issues: (i) How can we effectively tackle the issue of missing data in tabular datasets, as exemplified by the data missingness detailed in **Table 1**? (ii) How can the robustness of the TabNet model’s predictive accuracy be enhanced to ensure reliable predictions, even in the face of fluctuations in individual indicators? (iii) How can the TabNet model be optimized to better mine and utilize the inherent associations within data features? As **Table 1** demonstrates, common test indicators can be naturally grouped into different feature sets based on the bodily functions they assess. Applying heuristic information from these sets not only has the potential to enhance the model’s predictive power but also provides a basis for set-level analysis and interpretation of the significance of various features. This aspect is crucial for a deeper understanding of the model’s decision-making process and for improving its applicability in clinical settings.

In the following section, we will delve into a detailed exposition of how we build upon the standard TabNet to construct our Group-informed Attentive Framework for Diabetes Mellitus Progression Prediction (GADMP) model. This discussion will encompass the methodologies employed to adapt the TabNet architecture to our specific research context.

## 3 Methodology

**Figure 1:**
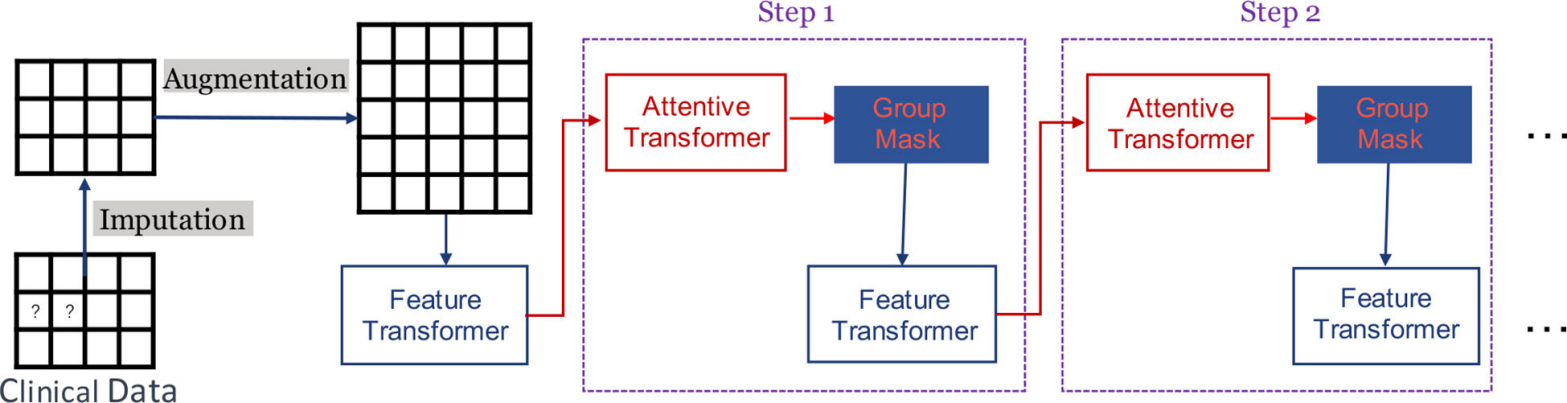
Graphical representation of DM progression prediction.

**Figure 1** provides a succinct depiction of our GADMP model, which is built upon TabNet. As illustrated, TabNet employs a unique multi-step architecture, where each step processes a distinct subset of features and makes individual decisions that cumulatively contribute to the final prediction. The Feature Transformer, a core component of TabNet, transforms the input features through a series of learnable, non-linear transformations, enabling the model to uncover intricate patterns and interactions. Additionally, the Attentive Transformer applies an attention mechanism to selectively focus on the most relevant features at each step, using learned attention masks that dynamically highlight and prioritize specific features based on their task relevance. In **Figure 1**, the components of Imputation, Augmentation, and Group Mask specifically address the three issues we previously mentioned. Imputation tackles the challenge of missing data, Augmentation enhances the robustness of the model against fluctuations in individual indicators, and Group Mask leverages the inherent associations within data features, aligning with the natural groupings of common test indicators. These enhancements to the standard TabNet architecture are pivotal in tailoring our GADMP model to effectively predict diabetes progression, ensuring both high accuracy and interpretability.

### 3.1 Imputation

In related work, two primary methods have been employed to address the issue of missing features: one approach involves filling in missing values with the mean or mode of the corresponding columns in the training dataset Jerez et al. (36) Neves et al. (37), while the other leverages machine learning algorithms, such as Random Forest or k-Nearest Neighbors, to predict these missing values Pantanowitz and Marwala (38) Tang and Ishwaran (39). However, given the severity of missing features in medical examination data, as evidenced in **Table 1**, relying on simplistic mechanisms or algorithms for data imputation can be ineffective under the constraint of limited known information, potentially impacting the accuracy of target outcome predictions.

In this work, we adopt a group information-inspired multi-stage strategy to achieve as accurate data imputation as possible. Our approach is as follows: (i) We use feature columns with less significant missing phenomena in tabular data (i.e., the 19 features in **Table 1**: *A*_1_, *A*_2_, *F*_1_ − *F*_12_, and *G*1 − *G*5) as clustering features and apply the DBSCAN algorithm Ester et al. (40) to divide all patients into different groups; (ii) For each patient, we calculate the mean values of all other features (excluding clustering features) within their group to create *m* additional cluster-level features (averaged indicators of patients in the corresponding cluster); (iii) Using these augmented features and the existing non-missing feature values, we progressively predict missing values using the LightGBM algorithm Ke et al. (28). Specifically, we first predict the missing values of the *B*_1_ feature, then use the predicted *B*_1_ values as known information to predict the next set of missing values, and continue this process until all missing feature values have been predicted.

We answer two key questions to elucidate the motivation behind adopting a group information-inspired multi-stage strategy for missing data imputation: (i) Why is clustering performed initially? In medical examination cohorts, individuals within the same group often exhibit similarities in various indicators Sakib et al. (41) Wahlqvist et al. (42). This implies that the missing feature values of a patient in a group might be similar to the corresponding indicators of other patients in the same group. Therefore, we utilize the feature values of corresponding feature columns within the group as additional information, providing a more comprehensive basis for predicting missing values. (ii) Why predict missing values in a stepwise manner? As **Table 1** shows, there is a tendency for missing data to occur across all tests within the same category, likely because patients tend to choose tests based on the bodily functions that these tests are intended to assess, leading to entire categories (like Lipid Profile, feature group *D*) being skipped. Directly using a unified model to predict multiple missing indicators for a patient might not yield optimal results. Hence, we adopt a stepwise filling approach, where each prediction cycle uses the values predicted in the previous cycle as known information to build a new model for predicting the next missing indicator.

Our strategy for missing data imputation employs the DBSCAN clustering algorithm and the LightGBM prediction algorithm. This choice is driven by: (i) DBSCAN’s ability to cluster based on data point density, automatically determine the number of clusters, robustness to noise, and suitability for tabular data; (ii) LightGBM’s efficiency in handling complex relationships and large-scale data, particularly apt for datasets with numerous features and in scenarios with missing data. The results of our experiments further validate the effectiveness of our proposed group information-inspired multi-stage data imputation strategy.

### 3.2 Augmentation

To enhance the robustness of predictions for tabular data, existing work has primarily focused on the design of machine learning Shah and Pradhan (43) Kudari (44) or deep learning algorithms Rajkomar et al. (45) Somepalli et al. (46), with a lack of solutions targeting underlying factors at the data level. Considering the unique nature of medical examination indicators, where patients’ metrics can fluctuate within a certain range, fluctuations in individual indicators at the data level can fundamentally affect the predictions of a trained model, thereby impacting its robustness. In this work, we propose a dual-layer data augmentation approach, encompassing both sample augmentation and feature augmentation, to further enhance the robustness of the TabNet model in predicting blood glucose levels.

Sample augmentation involves adding random perturbation values $\Delta_{f}$ to specific features *f* of a patient, where −ϵf < Δf < ϵf. This method allows us to create augmented samples, expanding the training environment and enhancing the model’s robustness against fluctuations in indicators. Feature augmentation involves adding group-level features to each sample. Assuming that samples within the same group share similar test indicator values to some extent, incorporating group-level features can help mitigate the impact of minor fluctuations in individual indicators within the group on the model. During the augmentation process, all features are used as clustering features, followed by clustering using the DBSCAN algorithm. We then select specific feature sets within each group and use their averaged indicator values to augment the feature columns of every patient in the group. This two-pronged augmentation strategy is designed to bolster the model’s resilience to variations in data, thereby improving its overall predictive robustness.

In practical implementation, for sample augmentation, we randomly select *n* features (perturbed features) from the feature columns (excluding gender and age) and add perturbation to them. The corresponding ϵf is set to one-twentieth (1/20) of the average value of each selected feature column. For each patient, we generate two augmented samples. The experimental results indicate that our proposed augmentation method not only bolsters the robustness of the TabNet model but also enhances its predictive accuracy. This strategy effectively counters the inherent variability in medical data, ensuring that the model remains reliable and precise, even in the presence of individual feature value fluctuations. By introducing controlled variability through augmentation, the model is trained to be more resilient to the variations commonly encountered in real-world data, thereby improving its applicability and effectiveness in clinical scenarios.

Current aggregation methods in diabetes management, such as those proposed by Sampath et al. Sampath et al. (47), primarily focus on combining glycemic control indices like the Low Blood Glucose Index (LBGI) to predict outcomes such as nocturnal hypoglycemia. These methods typically utilize linear combinations of individual glycemic indices derived from daily glucose measurements to enhance prediction accuracy. In contrast, our approach significantly advances these methodologies by integrating a more diverse array of clinical data across various categories. This broader spectrum of data allows our model to capture complex interactions between different health indicators, enhancing its applicability to various diabetes progression scenarios. Our innovative use of a deep attentive transformer to integrate features at an earlier stage and dynamically weigh their importance marks a substantial improvement over the traditional linear aggregation methods. Furthermore, we proactively address the challenge of missing data with sophisticated imputation strategies, ensuring robust performance across datasets with incomplete information. This comprehensive and advanced approach not only aligns with but also significantly advances current methodologies, offering a more effective tool for diabetes management in clinical settings.

### 3.3 Group masking

In the standard TabNet model, individual-level feature masking is typically employed during the modeling process to learn and interpret the importance of each independent feature for model predictions. This individual-level masking mechanism has achieved commendable results in various prediction and classification tasks Arik and Pfister (22). However, for tasks like predicting diabetes progression based on physical examination and medical test indicators, this approach might actually impede model effectiveness. This is because medical test indicators, corresponding to different bodily functions, are naturally divided into distinct sets. Indicators within the same set, indicating the same bodily function, tend to have similar effects on diabetes progression prediction. Individual-level feature masking could lead to inconsistencies in the importance assigned to indicators within the same set, potentially causing conflicts in model predictions.

Therefore, in this paper, we explore the uniform impact of the same group of indicators on the model, that is, applying the same mask to features within the same set (group), guiding the model’s learning process. This group masking strategy prevents the model from overly relying on any single feature during training, thereby enhancing its generalization capability. Additionally, it allows the model to perceive heuristic information between feature groups, further leveraging the natural and tight correlations among features to improve the model’s predictive performance. This approach not only addresses the limitations of individual-level masking in the context of diabetes progression prediction but also harnesses the collective strength of feature groups to achieve more accurate and reliable predictions.

During our experimental process, we selectively applied group masking to different sets of medical test indicators. This approach allowed us to further analyze and summarize the impact of various bodily function indicators on the progression of diabetes. By implementing group-level masking, we aimed to facilitate researchers in understanding the influence of different bodily functions on diabetes at a group level. This method not only enhances the interpretability of the model but also provides valuable insights into how different sets of indicators, each representing specific bodily functions, contribute to the overall prediction of diabetes progression. Such an approach is instrumental in advancing the understanding of diabetes from a more holistic and function-oriented perspective.

## 4 Experiments

We split the data into 80%, 10%, and 10% for training, validation, and test splits, respectively. For all experiments, we use the same training, validation and testing data. The test performance reported in this work of all compared methods is based on peak validation results.

In this study, we employ a comprehensive set of competitive baselines to ensure a robust comparison and evaluation of our model’s performance. Specifically, we utilize Standard TabNet Arik and Pfister (22), a deep learning-based approach designed for tabular data; LightGBM Ke et al. (28) and XGBoost Chen and Guestrin (27), both of which are highly efficient gradient boosting frameworks known for their speed and accuracy; MLP (Multi-Layer Perceptron), a classic type of neural network from Mocanu et al. (48) used in numerous deep learning tasks; and Adaptive Neural Trees Tanno et al. (49), a powerful ensemble technique that combines multiple weak prediction models to form a strong predictor. The selection of these diverse and well-established baselines allows for a thorough and fair assessment of our model’s capabilities in comparison to the current state-of-the-art methods.

### 4.1 Evaluation metrics

In evaluating the performance of all compared methods, we employ two standard metrics: Mean Absolute Error (MAE), Mean Squared Error (MSE), and Root Mean Squared Error (RMSE). MAE is defined as the average of the absolute differences between the predicted and actual values, mathematically represented as $\text{MAE }=\frac{1}{n}\sum_{i=1}^{n}\left|y_{i}-\hat{y_{i}}\right|$ , where *y_i_* and *y_i_* denote the actual and predicted values, respectively, and *n* is the number of observations. This metric is particularly useful for its interpretability and robustness to outliers. On the other hand, MSE, defined as $MAE\;=\frac{1}{n}{\sum_{i=1}^{n}\left(y_{i}-\hat{y_{i}}\right)}^{2}$ , provides a measure that penalizes larger errors more severely by squaring the differences between predicted and actual values. RMSE, given as $RMSE\;=\sqrt{\frac{1}{n}\sum i=1^{n}{\left(y_{i}-\hat{y_{i}}\right)}^{2}}$ , is essentially the square root of MSE and provides a more interpretable scale of error magnitude. The employment of these metrics facilitates a thorough assessment of model accuracy, considering both the magnitude of errors and the significance of larger deviations in predictions.

The aforementioned metrics (i.e., MAE, MSE, and RMSE) are selected for their ability to quantify the precision of blood glucose predictions at specific time points. While our approach focuses on the accuracy of predictions rather than tracking diabetes progression over time, these metrics are critical for assessing the current state of diabetes in patients. High accuracy in these predictions can indicate stability, improvement, or deterioration in the patient’s diabetic condition, which is crucial for effective disease management. By reliably determining the state of diabetes at each measured point, these metrics facilitate informed clinical decisions, helping to tailor interventions and adjust treatment strategies.

### 4.2 Results and analysis

In this section, we conduct a comprehensive analysis and comparison on the method we proposed from multiple perspectives.

#### 4.2.1 Cluster analysis

**Figure 2:**
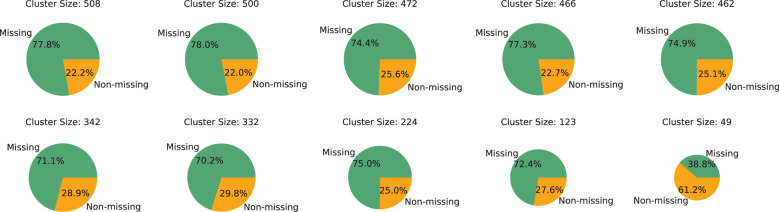
Proportional distribution of samples with missing features across different clusters.

Prior to engaging in experimental comparisons, an initial analysis of the data’s group dynamics is conducted. Utilizing 19 features, as mentioned in Section 3.1, as clustering attributes, the DBSCAN algorithm is applied to cluster patients, resulting in the formation of 19 distinct groups. These groups are then ordered by size, and a detailed analysis is conducted on the five largest and five smallest groups, the results of which are presented in **Figure 2**. The analysis reveals that, with the exception of the smallest group, the majority of samples in each group has missing features, highlighting the severity of the data missingness issue. Furthermore, it is observed that at least 20% of patients in each group possessed

complete feature columns. This finding indicates that, despite the significant data missingness, it is still feasible to utilize the samples with complete medical test indicators within each group to provide additional group-level insights for samples with missing features. This approach can potentially aid in more accurately predicting missing information, leveraging the available complete data within the groups to mitigate the challenges posed by data incompleteness.

#### 4.2.2 Performance comparison

The experimental results for diabetes progression prediction (using blood glucose concentration as an example) are presented in **Table 2** with respect to three evaluation metrics. The best-performing results are highlighted in bold. It is evident that our proposed group-informed attentive framework for diabetes mellitus progression prediction GADMP method achieves the most accurate performance across all three metrics. Even when compared with multiple state-of-the-art baselines, our method demonstrates significant improvements, underscoring its superiority. This indicates that the GADMP not only effectively addresses the challenges inherent in diabetes progression prediction but also sets a new benchmark in terms of accuracy and reliability in this domain.

The results presented in **Table 2** are the averages of multiple experimental runs. In our experiments, we initially utilized grid search techniques to explore various configurations and found that 23 cluster features and 8 perturbed features provided optimal results. However, the primary focus of our research is to demonstrate how cluster-level features effectively address issues of missing data, and how sample augmentation enhances model robustness. Therefore, to better highlight the model’s generalizability and practical application, we standardized the number of cluster features at 20 (*m*) and perturbed features at 10 (*n*). This decision ensures our results emphasize the utility of our approach across different scenarios, rather than delving into detailed feature optimization. This standardization supports our goal of showcasing the broad applicability and effectiveness of our model.

**Table 2:** Performance comparison.

Method	MSE	MAE	RMSE
MLP	1.639	0.807	1.295
LightGBM	1.630	0.790	1.286
XGBoost	1.619	0.765	1.279
Adaptive Neural Trees	1.625	0.781	1.284
Standard TabNet	1.614	0.756	1.269
GADMP	**1.589**	**0.714**	**1.260**

The bold value indicates the best prediction result w.r.t. a specific evaluation metric.

#### 4.2.3 Ablation study

**Figure 3:**
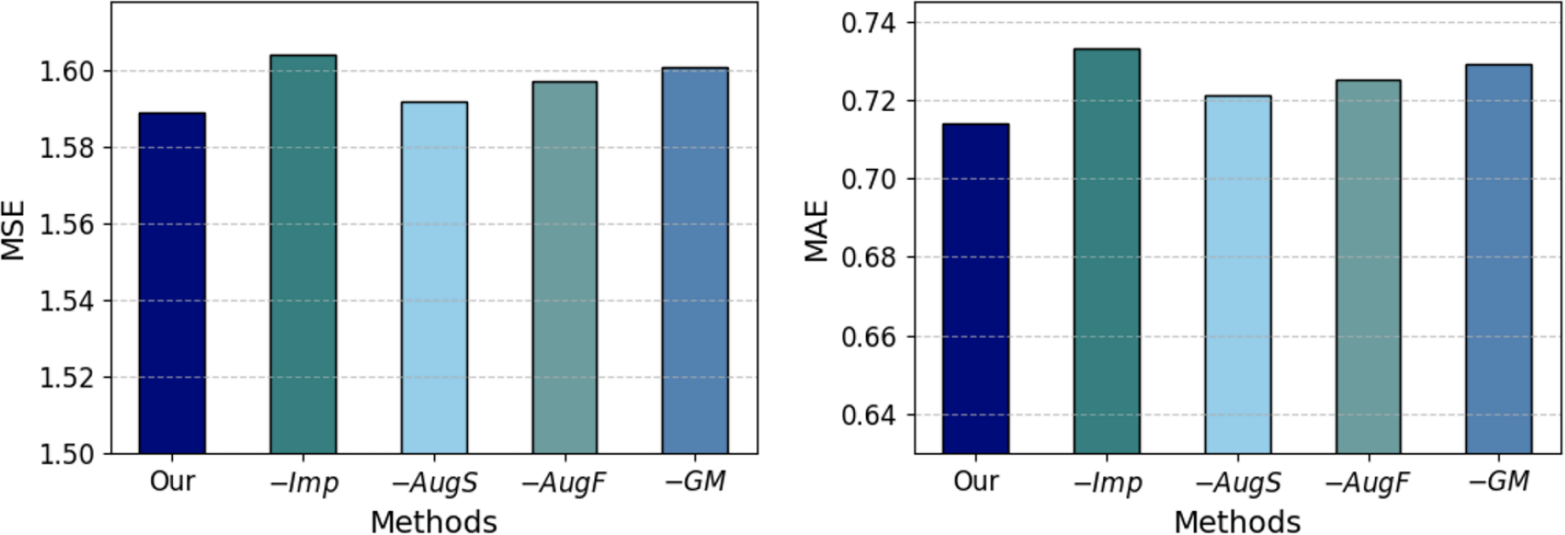
Results of ablation experiments.

To validate the contribution of different modules of our method to diabetes progression prediction, we conduct an ablation study, the results of which are displayed in **Figure 3**. The variants ‘Our’, ‘−*Imp*’, ‘−*AugS*’, ‘−*AugF*’, and ‘−*GM*’ represent our complete group-informed approach, the removal of grouplevel features-aided imputation, the exclusion of sample augmentation, the omission of group-level feature augmentation, and the exclusion of group masking, respectively. The comparative results of these variant methods demonstrate that the full GADMP model achieve the lowest prediction error (highest accuracy). The increase in error rates upon removing different functional components indicates the usefulness of the various strategies proposed in the GADMP model for predicting diabetes progression. Notably, the removal of group-level features-aided imputation and the exclusion of group masking have the most significant

impact on the model’s predictive performance. This further underscores the significant importance of group-level features in predicting missing values and the role of group masking in uncovering the interrelationships among medical test indicators.

#### 4.2.4 Robustness study

We assess the robustness of the model by randomly adding permutations to the indicator features of test samples and then comparing the predictions with those of the original samples. The experimental results are displayed in **Table 3**, where the subscript of each original sample’s prediction value indicates the change in the experimental results after adding permutation Δ_f_ to 10 randomly selected continuous numerical features. It is observed that, even with fluctuations in the test sample data, the complete GADMP model demonstrated the most stable experimental outcomes compared to the variants and standard TabNet, while the variant without sample augmentation is most affected. This comparative result aligns with the rationale behind our implementation of sample augmentation and further validates the robustness of our model. The ability of GADMP to maintain consistent performance under data variability highlights its efficacy and reliability in real-world scenarios.

**Table 3:** Robustness performance comparison.

Method	MSE	MAE	RMSE
GADMP	1.589_+0.006_	0.714_+0.013_	1.260_+0.004_
GADMP−*AugS*	1.592_+0.013_	0.721_+0.021_	1.260_+0.009_
GADMP−*AugF*	1.597_+0.009_	0.725_+0.018_	1.260_+0.008_
TabNet	1.614_+0.016_	0.756_+0.028_	1.269_+0.013_

Aligning with **Figure 3** and **Table 3**, we can find that the integration of group-level features significantly improved the predictive accuracy and robustness of our model. This enhancement is primarily due to the rich biological context and group-level perspectives these features provide, allowing the model to capitalize on interconnected physiological processes. Additionally, the sample augmentation process where perturbations are introduced to randomly selected features has proven essential in further enhancing the robustness and effectiveness of our model, preparing it to handle real-world clinical variations effectively. These modifications collectively ensure that our framework not only achieves high accuracy but also maintains consistent performance across varied and unpredictable clinical settings.

#### 4.2.5 Importance analysis

The feature selection masks of standard TabNet can shed light on the selected features at each step. If M_b,j_[i]=0, then *j^th^* feature of the *b^th^* sample should have no contribution to the decision. TabNet aims to quantify an aggregate feature importance in addition to analysis of each step. Combining the masks at different steps requires a coefficient that can weigh the relative importance of each step in the decision.

In our practical experiments, two important discoveries are made: (i) After clustering all samples, we find that adding group-level features of all numerical features as augmented features (i.e., adding 37 additional features for each sample) does not yield optimal results. This is because some test indicators inherently have a minimal impact on blood glucose concentration prediction. Adding their corresponding group-level features can inadvertently amplify their influence on the model, ultimately diminishing its effectiveness. Through multiple comparative cross-experiments, we discover that the best results were achieved by adding group-level features of Liver Function Tests indicators (*B*_1_-*B*_8_) and Lipid Profile indicators (*D*_1_-*D*_4_) as augmented features. The superior experimental outcomes we report are obtained after incorporating these 12 group-level augmented features; (ii) Our experimental data comprise seven groups of indicators, and the specific results indicate that applying group masking to all seven indicator groups, *i.e.*, using the same mask for indicators of a group, does not lead to optimal outcomes. This may be due to the varying importance of different groups of features in model prediction. Applying group masking to an indicator group with a weak correlation to diabetes progression might inadvertently increase its influence on the model, thereby negatively impacting prediction accuracy. After multiple experiments, we find that the highest prediction accuracy is achieved when group masking is applied to Liver Function Tests and Lipid Profile indicators. These findings indirectly highlight the significance of Liver Function Tests and Lipid Profile indicators in influencing the progression of diabetes.

**Figure 4:**
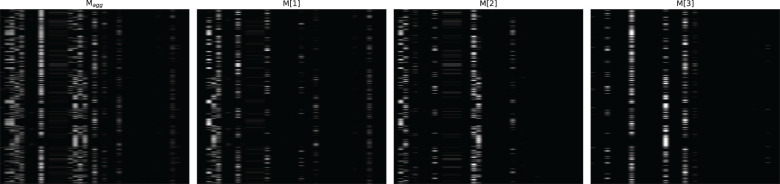
Feature importance heatmap of our model without group mask nor group-level augmented features. Masks M[i] (that indicate feature selection at *i*th step) and the aggregate feature importance mask M*_agg_* showing the global instance-wise feature selection.

**Figure 5:**
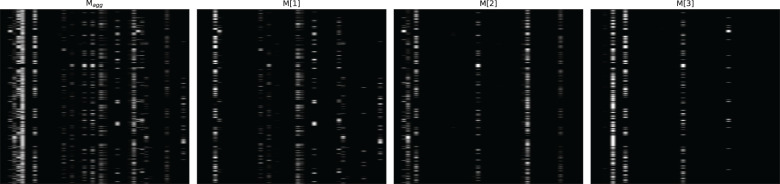
Feature importance heatmap of our model using augmented features without group mask.

**Figure 6:**
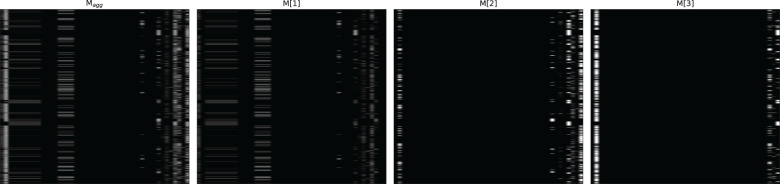
Feature importance heatmap of our model using group mask and group-level augmented features.

**Figures 4**–**6** respectively illustrate the feature importance of GADMP without group masking nor augmented features, GADMP using augmented features without group mask, and GADMP with both group masking and augmented features. A comparison between **Figures 4**, **5** reveals that, in the absence of augmented features and group masking (**Figure 4**), the model’s learned mask values are relatively dispersed, indicating that it has not effectively identified features significantly correlated with diabetes progression. However, upon incorporating group-level augmented features (**Figure 5**), the model increasingly emphasizes the impact of certain indicators within Liver Function Tests, suggesting that the addition of corresponding group augmented features has enabled the model to recognize the importance of the indicators in this group. This leads to two important findings: (i) Liver Function Tests indicators are closely related to diabetes progression, aligning with several existing medical research works Cho et al. (50) Leeds et al. (51) Ni et al. (52). Our work further validates this relationship through the attentive deep learning prediction model and a group-informed approach; (ii) The group-level augmented features we proposed are genuinely beneficial for the model’s predictions, demonstrating their inherent importance (as seen in the first step in **Figure 4**) and enhancing the impact of related individual indicators.

The comparison of **Figure 6** with **Figures 4** and **Figure 5** shows significant differences in mask importance when group masking and augmented features are applied: (i) Indicators of Liver Function Tests and Lipid Profile exhibit a unified importance in model predictions; (ii) Lipid Profile indicators, known to significantly impact diabetes progression Arora et al. (53) Uttra et al. (54) Artha et al. (55), are effectively identified for the first time; (iii) The importance of augmented features of Lipid Profile and Liver Function Tests is further reinforced. These comparative findings underscore the tight correlation between Lipid Profile and Liver Function Tests with diabetes progression and their significant influence on model predictions.

## 5 Conclusion

In conclusion, our Group-Informed Attentive Framework for Diabetes Mellitus Progression Prediction (GADMP) model represents a novel and effective approach in the realm of medical research, particularly in the prediction of diabetes progression. The model’s innovative design, which incorporates group-level augmented features and group masking, has demonstrated significant improvements in predictive accuracy and robustness, as evidenced by our comprehensive experimental results.

The key findings from our experiments highlight the critical role of Liver Function Tests and Lipid Profile indicators in diabetes progression. By integrating these specific indicators into our model through group-level augmentation and masking, we have not only enhanced the model’s predictive capabilities but also provided new insights into the complex relationships between these medical indicators and diabetes progression. This approach has proven to be particularly effective in identifying and emphasizing the importance of these indicators, which aligns with and extends existing medical research.

Furthermore, the GADMP model’s ability to handle data variability and missingness through its advanced augmentation strategies significantly contributes to its practical applicability in clinical settings. The model’s performance in the presence of data fluctuations underscores its reliability and potential as a tool for healthcare professionals in managing and predicting diabetes progression.

Overall, the GADMP model stands as a testament to the potential of deep learning in medical research, offering a promising direction for future studies in diabetes and other related medical fields. Its success in accurately predicting diabetes progression paves the way for more personalized and effective treatment strategies, ultimately contributing to better patient outcomes and advancements in healthcare.

## Data availability statement

Publicly available datasets were analyzed in this study. This data can be found here: https://tianchi.aliyun.com/competition.

## Author contributions

CS: Conceptualization, Methodology, Project administration, Validation, Writing – original draft, Writing – review & editing. RY: Methodology, Writing – review & editing.

## Footnotes

1
https://tianchi.aliyun.com/competition/entrance/231638/information.

## References

[1] Association AD . Diagnosis and classification of diabetes mellitus. Diabetes Care. (2010) 33:S62–9. doi: 10.2337/dc10-S062 PMC2797383 20042775

[2] Alam U Asghar O Azmi S Malik RA . General aspects of diabetes mellitus. Handb Clin Neurol. (2014) 126:211–22. doi: 10.1016/B978-0-444-53480-4.00015-1 25410224

[3] Tomic D Shaw JE Magliano DJ . The burden and risks of emerging complications of diabetes mellitus. Nat Rev Endocrinol. (2022) 18:525–39. doi: 10.1038/s41574-022-00690-7 PMC9169030 35668219

[4] Nathan DM . Long-term complications of diabetes mellitus. New Engl J Med. (1993) 328:1676–85. doi: 10.1056/NEJM199306103282306 8487827

[5] Cole JB Florez JC . Genetics of diabetes mellitus and diabetes complications. Nat Rev Nephrol. (2020) 16:377–90. doi: 10.1038/s41581-020-0278-5 PMC9639302 32398868

[6] Litjens G Sánchez CI Timofeeva N Hermsen M Nagtegaal I Kovacs I . Deep learning as a tool for increased accuracy and efficiency of histopathological diagnosis. Sci Rep. (2016) 6:26286. doi: 10.1038/srep26286 27212078 PMC4876324

[7] Ayon SI Islam MM . Diabetes prediction: a deep learning approach. Int J Inf Eng Electronic Business. (2019) 12:21. doi: 10.5815/ijieeb.2019.02.03

[8] Liu Y Jain A Eng C Way DH Lee K Bui P . A deep learning system for differential diagnosis of skin diseases. Nat Med. (2020) 26:900–8. doi: 10.1038/s41591-020-0842-3 32424212

[9] Pal S Mishra N Bhushan M Kholiya PS Rana M Negi A . (2022). Deep learning techniques for prediction and diagnosis of diabetes mellitus, in: 2022 International mobile and embedded technology conference (MECON), IEEE. pp. 588–93.

[10] Arcadu F Benmansour F Maunz A Willis J Haskova Z Prunotto M . Deep learning algorithm predicts diabetic retinopathy progression in individual patients. NPJ digital Med. (2019) 2:92. doi: 10.1038/s41746-019-0172-3 PMC6754451 31552296

[11] Ljubic B Hai AA Stanojevic M Diaz W Polimac D Pavlovski M . Predicting complications of diabetes mellitus using advanced machine learning algorithms. J Am Med Inf Assoc. (2020) 27:1343–51. doi: 10.1093/jamia/ocaa120 PMC7647294 32869093

[12] Refat MAR Al Amin M Kaushal C Yeasmin MN Islam MK . (2021). A comparative analysis of early stage diabetes prediction using machine learning and deep learning approach, in: 2021 6th International Conference on Signal Processing, Computing and Control (ISPCC), IEEE. pp. 654–9.

[13] Gupta H Varshney H Sharma TK Pachauri N Verma OP . Comparative performance analysis of quantum machine learning with deep learning for diabetes prediction. Complex Intelligent Syst. (2022) 8:3073–87. doi: 10.1007/s40747-021-00398-7

[14] Zhu T Li K Herrero P Chen J Georgiou P . A deep learning algorithm for personalized blood glucose prediction. KHD@ IJCAI. (2018), 64–78.

[15] Freiburghaus J Rizzotti A Albertetti F . (2020). A deep learning approach for blood glucose prediction of type 1 diabetes, in: Proceedings of the Proceedings of the 5th International Workshop on Knowledge Discovery in Healthcare Data co-located with 24th European Conference on Artificial Intelligence (ECAI 2020), 29–30 August 2020, Santiago de Compostela, Spain, 29–30 August 2020.

[16] Zhu T Kuang L Piao C Zeng J Li K Georgiou P . Population-specific glucose prediction in diabetes care with transformer-based deep learning on the edge. IEEE Trans Biomed Circuits Syst. (2024) 1–12. doi: 10.1109/TBCAS.2023.3348844 38163299

[17] Yahyaoui A Jamil A Rasheed J Yesiltepe M . (2019). A decision support system for diabetes prediction using machine learning and deep learning techniques. In 1st International Informatics and Software Engineering Conference: Innovative Technologies for Digital Transformation, IISEC 2019-Proceedings. Institute of Electrical and Electronics Engineers Inc.

[18] Choi SB Kim WJ Yoo TK Park JS Chung JW Lee Y-h . Screening for prediabetes using machine learning models. In: Computational and mathematical methods in medicine (2014), 618976. 10.1155/2014/618976 PMC4140121 25165484

[19] Li L Cheng Y Ji W Liu M Hu Z Yang Y . Machine learning for predicting diabetes risk in western China adults. Diabetol Metab Syndrome. (2023) 15:165. doi: 10.1186/s13098-023-01112-y PMC10373320 37501094

[20] Pala V Sieri S Masala G Palli D Panico S Vineis P . Associations between dietary pattern and lifestyle, anthropometry and other health indicators in the elderly participants of the epic-Italy cohort. Nutrition Metab Cardiovasc Dis. (2006) 16:186–201. doi: 10.1016/j.numecd.2005.05.009 16580586

[21] Du Y Dennis B Rhodes SL Sia M Ko J Jiwani R . Technology-assisted self-monitoring of lifestyle behaviors and health indicators in diabetes: qualitative study. JMIR Diabetes. (2020) 5:e21183. doi: 10.2196/21183 32857056 PMC7486673

[22] Arik SÖ. Pfister T . (2021). Tabnet: Attentive interpretable tabular learning, in: Proceedings of the AAAI conference on artificial intelligence, Association for the Advancement of Artificial Intelligence (AAAI). Vol. 35. pp. 6679–87.

[23] Sherwani SI Khan HA Ekhzaimy A Masood A Sakharkar MK . Significance of hba1c test in diagnosis and prognosis of diabetic patients. biomark Insights. (2016) 11:BMI–S38440. doi: 10.4137/BMI.S38440 PMC4933534 27398023

[24] Weykamp C . Hba1c: a review of analytical and clinical aspects. Ann Lab Med. (2013) 33:393. doi: 10.3343/alm.2013.33.6.393 24205486 PMC3819436

[25] Svetnik V Liaw A Tong C Culberson JC Sheridan RP Feuston BP . Random forest: a classification and regression tool for compound classification and qsar modeling. J Chem Inf Comput Sci. (2003) 43:1947–58. doi: 10.1021/ci034160g 14632445

[26] Friedman JH . Greedy function approximation: a gradient boosting machine. Ann Stat. (2001) 29.2:1189–232. doi: 10.1214/aos/1013203451

[27] Chen T Guestrin C . (2016). Xgboost: A scalable tree boosting system, in: Proceedings of the 22nd acm sigkdd international conference on knowledge discovery and data mining, pp. 785–94.

[28] Ke G Meng Q Finley T Wang T Chen W Ma W . Lightgbm: A highly efficient gradient boosting decision tree. In: Proceedings of the 31st International Conference on Neural Information Processing Systems. (2017), 3149–57.

[29] Nguyen HV Byeon H . Prediction of out-of-hospital cardiac arrest survival outcomes using a hybrid agnostic explanation tabnet model. Mathematics. (2023) 11:2030. doi: 10.3390/math11092030

[30] Shwartz-Ziv R Armon A . Tabular data: Deep learning is not all you need. Inf Fusion. (2022) 81:84–90. doi: 10.1016/j.inffus.2021.11.011

[31] Shen D Wu G Suk H-I . Deep learning in medical image analysis. Annu Rev Biomed Eng. (2017) 19:221–48. doi: 10.1146/annurev-bioeng-071516-044442 PMC5479722 28301734

[32] Chatterjee A Gupta U Chinnakotla MK Srikanth R Galley M Agrawal P . Understanding emotions in text using deep learning and big data. Comput Hum Behav. (2019) 93:309–17. doi: 10.1016/j.chb.2018.12.029

[33] Purwins H Li B Virtanen T Schlüter J Chang S-Y Sainath T . Deep learning for audio signal processing. IEEE J Selected Topics Signal Process. (2019) 13:206–19. doi: 10.1109/JSTSP.2019.2908700

[34] Yan J Xu T Yu Y Xu H . Rainfall forecast model based on the tabnet model. Water. (2021) 13:1272. doi: 10.3390/w13091272

[35] Chen Y Li H Dou H Wen H Dong Y . Prediction and visual analysis of food safety risk based on tabnet-gra. Foods. (2023) 12:3113. doi: 10.3390/foods12163113 37628112 PMC10453234

[36] Jerez JM Molina I García-Laencina PJ Alba E Ribelles N Martín M . Missing data imputation using statistical and machine learning methods in a real breast cancer problem. Artif Intell Med. (2010) 50:105–15. doi: 10.1016/j.artmed.2010.05.002 20638252

[37] Neves DT Alves J Naik MG Proenc¸ a,AJ Prasser F . From missing data imputation to data generation. J Comput Sci. (2022) 61:101640. doi: 10.1016/j.jocs.2022.101640

[38] Pantanowitz A Marwala T . Missing data imputation through the use of the random forest algorithm. In: Advances in computational intelligence. Springer (2009) 61:53–62.

[39] Tang F Ishwaran H . Random forest missing data algorithms. Stat Anal Data Mining: ASA Data Sci J. (2017) 10:363–77. doi: 10.1002/sam.11348 PMC5796790 29403567

[40] Ester M Kriegel H-P Sander J Xu X . A density-based algorithm for discovering clusters in large spatial databases with noise. In kdd. vol. (1996) 96:226–31.

[41] Sakib MN Shooshtari S St. John P Menec V . The prevalence of multimorbidity and associations with lifestyle factors among middle-aged canadians: an analysis of canadian longitudinal study on aging data. BMC Public Health. (2019) 19:1–13. doi: 10.1186/s12889-019-6567-x 30819126 PMC6394050

[42] Wahlqvist M Möller C Möller K Danermark B . Similarities and differences in health, social trust, and financial situation in people with usher syndrome, a bio-psychosocial perspective. Front Psychol. (2020) 11:1760. doi: 10.3389/fpsyg.2020.01760 32982818 PMC7485379

[43] Shah S Pradhan M . R-ga: An efficient method for predictive modeling of medical data using a combined approach of random forests and genetic algorithm. ICTACT J Soft Computing 6.2. (2016) 6. doi: 10.21917/ijsc.2016.0160

[44] Kudari JM . Data analytics to predict, detect, and monitor chronic autoimmune diseases using machine learning algorithms: Preventing diseases with the power of machine learning. In: Machine learning and data analytics for predicting, managing, and monitoring disease (IGI global) (2021), p. 150–82.

[45] Rajkomar A Oren E Chen K Dai AM Hajaj N Hardt M . Scalable and accurate deep learning with electronic health records. NPJ digital Med. (2018) 1:18. doi: 10.1038/s41746-018-0029-1 PMC6550175 31304302

[46] Somepalli G Goldblum M Schwarzschild A Bruss CB Goldstein T . Saint: Improved neural networks for tabular data *via* row attention and contrastive pre-training. arXiv. (2021).

[47] Sampath S Tkachenko P Renard E Pereverzev SV . Glycemic control indices and their aggregation in the prediction of nocturnal hypoglycemia from intermittent blood glucose measurements. J Diabetes Sci Technol. (2016) 10:1245–50. doi: 10.1177/1932296816670400 PMC5094347 27660190

[48] Mocanu DC Mocanu E Stone P Nguyen PH Gibescu M Liotta A . Scalable training of artificial neural networks with adaptive sparse connectivity inspired by network science. Nat Commun. (2018) 9:2383. doi: 10.1038/s41467-018-04316-3 29921910 PMC6008460

[49] Tanno R Arulkumaran K Alexander D Criminisi A Nori A . (2019). Adaptive neural trees. In 36th International Conference on Machine Learning, ICML 2019. Proceedings of Machine Learning Research. Vol. 97, pp. 6166–75.

[50] Cho NH Jang HC Choi SH Kim HR Lee HK Chan JC . Abnormal liver function test predicts type 2 diabetes: a community-based prospective study. Diabetes Care. (2007) 30:2566–8. doi: 10.2337/dc07-0106 17626893

[51] Leeds J Forman E Morley S Scott A Tesfaye S Sanders D . Abnormal liver function tests in patients with type 1 diabetes mellitus: prevalence, clinical correlations and underlying pathologies. Diabetic Med. (2009) 26:1235–41. doi: 10.1111/j.1464-5491.2009.02839.x 20002475

[52] Ni H Soe HHK Htet A . Determinants of abnormal liver function tests in diabetes patients in Myanmar. Int J Diabetes Res. (2012) 1:36–41. doi: 10.5923/j.diabetes.20120103.02

[53] Arora M Koley S Gupta S Sandhu J . A study on lipid profile and body fat in patients with diabetes mellitus. Anthropologist. (2007) 9:295–8. doi: 10.1080/09720073.2007.11891015

[54] Uttra KM Devrajani BR Shah SZA Devrajani T Das T Raza S . Lipid profile of patients with diabetes mellitus (a multidisciplinary study). World Appl Sci J. (2011) 12:1382–4.

[55] Artha IMJR Bhargah A Dharmawan NK Pande UW Triyana KA Mahariski PA . High level of individual lipid profile and lipid ratio as a predictive marker of poor glycemic control in type-2 diabetes mellitus. Vasc Health Risk Manage. (2019) 15:149–57. doi: 10.2147/VHRM.S209830 PMC6560183 31239693

